# Causal relationships between obesity-related anthropometric indicators and sepsis risk: a Mendelian-randomization study

**DOI:** 10.3389/fnut.2024.1433754

**Published:** 2024-09-23

**Authors:** Chuchu Zhang, Jiajia Ren, Xi Xu, Hua Lei, Guorong Deng, Jueheng Liu, Xiaoming Gao, Jiamei Li, Xiaochuang Wang, Gang Wang

**Affiliations:** ^1^Department of Critical Care Medicine, The Second Affiliated Hospital of Xi’an Jiaotong University, Xi’an, China; ^2^Key Laboratory of Surgical Critical Care and Life Support, Ministry of Education, Xi’an Jiaotong University, Xi’an, China

**Keywords:** anthropometric indicator, inflammation, Mendelian randomization, obesity, sepsis

## Abstract

**Background:**

Previous studies have reported an association between obesity and risk of sepsis. However, the results have been inconsistent, and no causal inference can be drawn from them. Therefore, we conducted a Mendelian-randomization (MR) study to investigate causal relationships between available obesity-related anthropometric indicators and sepsis risk.

**Methods:**

We performed MR analyses using genome-wide association study (GWAS) summary statistics on 14 anthropometric indicators [namely body mass index (BMI), waist and hip circumferences (WC, HC), basal metabolic rate (BMR), whole-body fat mass (WBFM), trunk fat mass (TFM), leg fat mass (LFM), arm fat mass (AFM), body fat percentage (BFP), whole-body fat-free mass (WBFFM), trunk fat-free mass (TFFM), leg fat-free mass (LFFM), arm fat-free mass (AFFM), and whole-body water mass (WBWM)], sepsis, critical care sepsis, and 28-day death due to sepsis from the UK Biobank and FinnGen cohort. The primary method of MR analysis was inverse variance-weighted average method. Sensitivity analyses, including heterogeneity and horizontal-pleiotropy tests, were conducted to assess the stability of the MR results. Additionally, we applied multiple-variable MR (MVMR) to evaluate the effect of BMI on the relationship between each anthropometric indicator and sepsis risk.

**Results:**

Our MR analysis demonstrated causal relationships between 14 anthropometric indicators and sepsis of different severities. After we adjusted for BMI, MVMR analyses indicated that WC, BMR, LFM, WBFFM, TFFM, AFFM, and WBWM remained significantly associated with the presence of sepsis (all *p* < 0.05). A sensitivity analysis confirmed the reliability of our MR results, and no significant horizontal pleiotropy was detected.

**Conclusion:**

This MR study revealed that increases in obesity-related anthropometric indicators had causal associations with a higher risk of sepsis, which might provide important insights for the identification of individuals at risk for sepsis in community and hospital settings.

## 1 Introduction

Sepsis is a life-threatening condition caused by dysregulated host response to severe systemic infection that contributes to the global burden of disease (1, 2). Given the enormous healthcare costs of sepsis, the World Health Organization (WHO) has prioritized it as a global health concern (3). Therefore, identifying potential risk factors for sepsis is imperative to implementing early-prevention strategies for at-risk individuals. Obesity, defined as excessive accumulation of body fat, is a complex multifactorial disease that presents a severe public health challenge worldwide and contributes significantly to disability and death in the WHO European Region (4). Observational and Mendelian-randomization (MR) studies have shown that obesity, as determined by body mass index (BMI), is independently associated with an increased risk of bloodstream infection or sepsis (5–10). However, subsequent observational studies found that waist circumference (WC), an alternative anthropometric indicator, might be a greater predictor of sepsis than BMI, probably due to its strong relationship to visceral adiposity (11, 12). In addition, despite its practicality in clinical settings, BMI fails to describe body fat composition accurately (13). Therefore, a more comprehensive description of obesity traits could help clinicians better evaluate the risks of various diseases.

Indeed, apart from BMI and WC, several additional anthropometric indicators have the potential to offer a comprehensive and precise description of obesity traits. They include basal metabolic rate (BMR), whole-body fat mass (WBFM), trunk fat mass (TFM), leg fat mass (LFM), arm fat mass (AFM), body fat percentage (BFP), whole-body fat-free mass (WBFFM), trunk fat-free mass (TFFM), leg fat-free mass (LFFM), arm fat-free mass (AFFM), and whole-body water mass (WBWM). However, evidence from either observational studies or randomized controlled trials (RCTs) clarifying the causal relationship between each of these indicators and sepsis risk is scarce, hindering a comprehensive understanding of how obesity affects the risk of sepsis.

MR is a causal-inference approach to evaluating the causality of risk factors for disease that relies on single-nucleotide polymorphisms (SNPs) as instrumental variables (IVs) (14). MR possesses significant strengths such as reducing potential confounders and avoiding reverse-causality issues (15). According to Mendel’s second law, alleles are randomly assigned at conception, which helps minimize the effects of potential confounders (16). Moreover, reverse causality is unlikely because genotypic inheritance always precedes disease onset. To the best of our knowledge, no observational or MR study has yet explored the effects of comprehensive anthropometric indicators on risk of sepsis. Therefore, we conducted this MR study to elucidate the causal associations of available anthropometric indicators with sepsis risk, which could facilitate early identification of individuals at risk of sepsis in community and hospital settings.

## 2 Methods

### 2.1 Study design

An MR study typically relies on three fundamental assumptions to ensure the reliability of the findings. The relevance assumption stipulates that SNPs are closely correlated with the exposures under investigation. The independent assumption posits that SNPs are independent of any potential confounders. Lastly, the exclusivity assumption states that SNPs influence outcomes through exposures, excluding other pathways (14). In line with these assumptions, we obtained suitable IVs from publicly available genome-wide association study (GWAS) datasets to conduct MR analysis, with the goal of assessing the association between 14 obesity-related anthropometric indicators and risk of sepsis.

**Figure 1:**
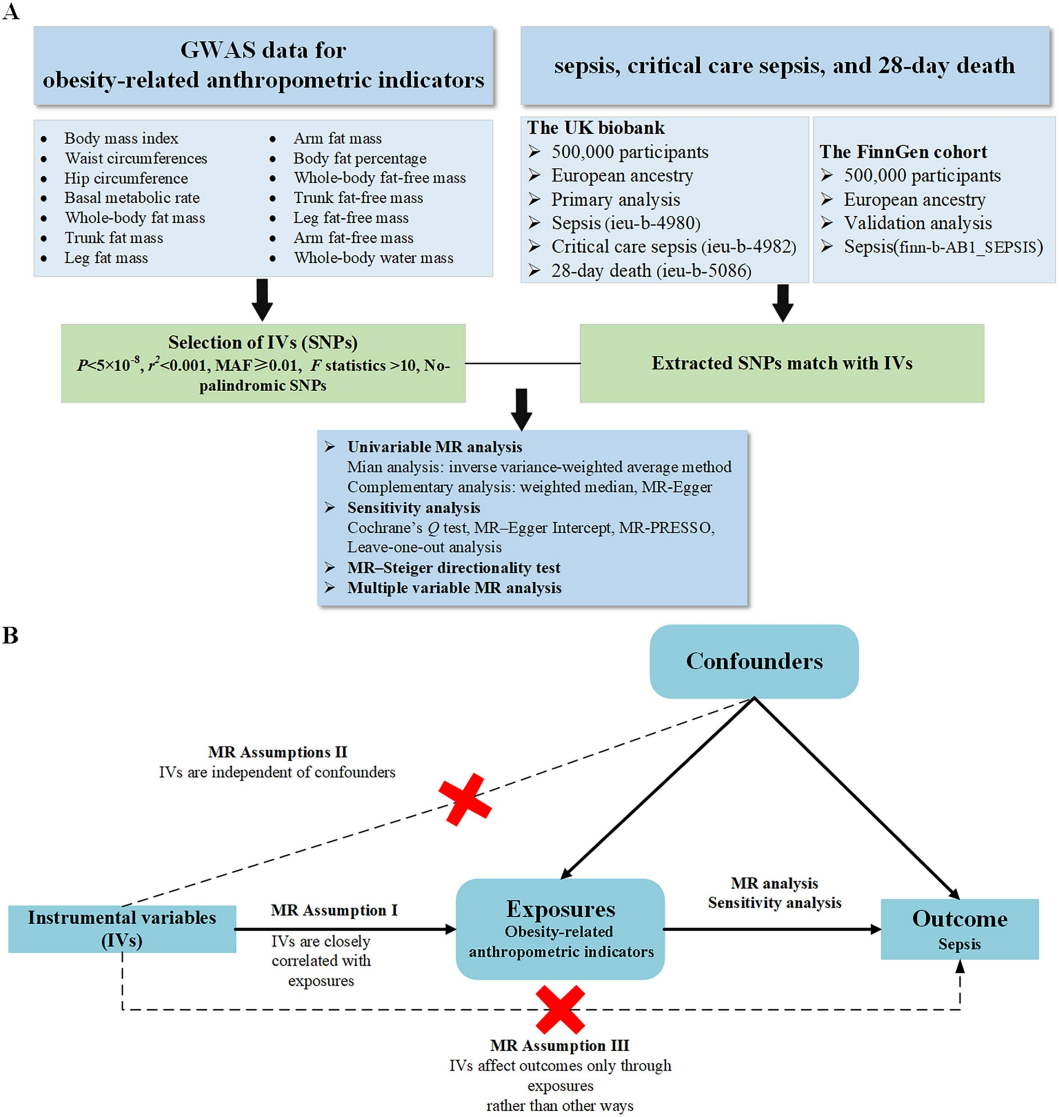
Overview of the design and analysis strategy of this Mendelian-randomization study **(A)**. The analysis process of this study **(B)**. Overview of the study design.

Our study used several methods for MR analysis, including the MR–Egger intercept test, weighted median, and inverse variance-weighted average method (IVW). In addition, we conducted the MR–Steiger directionality test to determine whether sepsis had a reverse-causal impact on body anthropometric indicators. To ensure the robustness of our results, we performed sensitivity analyses such as the heterogeneity test, the horizontal-pleiotropy test, and leave-one-out analysis. Finally, we also applied multiple-variable MR (MVMR) to examine whether the relationship between each indicator and sepsis risk was independent of BMI. The implementation of this study followed the STROBE-MR guideline (17), and the overall design was shown in Figure 1.

### 2.2 Data source

We obtained GWAS summary statistics on BMI (*N* = 681,275) from the Genetic Investigation of Anthropometric Traits consortium, an international collaboration aiming to identify genetic loci that modulate human body size and shape (18). Genetic IVs for anthropometric indicators, including hip circumference (HC; *N* = 462,117), WC (*N* = 462,166), BMR (*N* = 454,874), WBFM (*N* = 454,137), TFM (*N* = 454,588), LFM (*N* = 454,846), AFM (*N* = 454,757), BFP (*N* = 454,633), WBFFM (*N* = 454,850), TFFM (*N* = 454,508), LFFM (*N* = 454,835), AFFM (*N* = 454,753), and WBWM (*N* = 454,888), were initially sourced from the UK Biobank, a comprehensive biomedical database containing detailed genetic and health information from a sizeable cohort of individuals aged between 40 and 69 years (19). We measured the above-mentioned body composition indicators through whole-body bio-impedance analysis using the Tanita BC418MA body composition analyzer (Tanita Corporation of America, Inc., Arlington Heights, IL, United States) (20).

Summary-level GWAS data on sepsis were generated from the UK Biobank and the FinnGen cohort. The dataset from UK biobank was composed of 11,643 cases and 474,841 controls; and the validation dataset from the FinnGen cohort contained 6,164 cases and 197,660 controls. Sepsis was defined in accordance with International Classification of Disease-10 edition codes A02, A39, A40, and A41 (2). Furthermore, critical care sepsis and 28-day mortality due to sepsis were extracted from the UK Biobank, encompassing a total of 1,380 cases and 429,985 controls for critical care sepsis, as well as 1,896 cases and 484,588 controls for the assessment of sepsis-related death within a span of 28 days. Summary statistics were extracted exclusively from individuals of European descent to minimize potential confounding effects of race. All the above GWAS summary data can be downloaded or accessed online from the Integrative Epidemiology Unit Open GWAS Project.^1^ Table 1 provides an overview of the characteristic features of GWAS summary data for both obesity-related anthropometric indicators and sepsis-related outcomes.

**Table 1:** Characteristics of the GWAS summary data.

Exposures	GWAS ID	Consortium	Sample size	No. SNPs
Body mass index	ieu-b-40	GIANT	681,275	2,336,260
Hip circumference	ukb-b-15590	MRC-IEU	462,117	9,851,867
Waist circumference	ukb-b-9405	MRC-IEU	462,166	9,851,867
Basal metabolic rate	ukb-b-16446	MRC-IEU	454,874	9,851,867
Whole body fat mass	ukb-b-19393	MRC-IEU	454,137	9,851,867
Trunk fat mass	ukb-b-20044	MRC-IEU	454,588	9,851,867
Leg fat mass	ukb-b-18096	MRC-IEU	454,846	9,851,867
Arm fat mass	ukb-b-6704	MRC-IEU	454,757	9,851,867
Body fat percentage	ukb-b-8909	MRC-IEU	454,633	9,851,867
Whole body fat-free mass	ukb-b-13354	MRC-IEU	454,850	9,851,867
Trunk fat-free mass	ukb-b-17409	MRC-IEU	454,508	9,851,867
Leg fat-free mass	ukb-b-12828	MRC-IEU	454,835	9,851,867
Arm fat-free mass	ukb-b-19520	MRC-IEU	454,753	9,851,867
Whole body water mass	ukb-b-14540	MRC-IEU	454,888	9,851,867
Outcomes				
Sepsis	ieu-b-4980	UK Biobank	11,643 cases 474,841 controls	12,243,539
	finn-b-AB1_SEPSIS	FinnGen	6,164 cases 197,660 controls	16,380,410
Sepsis (critical care)	ieu-b-4982	UK Biobank	1,380 cases 429,985 controls	12,243,372
Sepsis (28 day death)	ieu-b-5086	UK Biobank	1,896 cases 484,588 controls	12,243,487

### 2.3 Instrumental-variable selection

In accordance with the three fundamental assumptions of the MR study, we established a set of screening criteria to identify applicable IVs associated with anthropometric indicators. We adopted a genome-wide significance threshold for SNP of *p* < 5 × 10^−8^ to satisfy the relevance assumption. SNPs with *r^2^* > 0.001 and physical distance <10,000 kb were filtered out to ensure absence of linkage disequilibrium (LD) (14). Next, SNPs with mismatched alleles, palindromic sequences, or minor allele frequencies (MAFs) < 0.01 were excluded. In addition, we ruled out SNPs that were either related to the outcome or not present in the outcome dataset. To assess IV strength, we calculated the *F* statistic for each SNP using the following formula (21):


$$F=r^{2}\times \left(N-2\right)/\left(1-r^{2}\right)$$

where *N* is the sample size of the exposure dataset, and *r^2^* is the proportion of variation explained by SNPs in the exposure data. The equation for determining *r^2^* is as follows (22):


$$r^{2}=2\times EAF\times \left(1-EAF\right)\times \beta^{2}/SD^{2}$$

where EAF is effect allele frequency, *β* is effect size, and *SD* is the standard deviation. Finally, we removed SNPs with *F* < 10 to mitigate bias associated with weak IVs (23). Characteristics of the selected SNPs are shown in Supplementary Table S1.

### 2.4 Statistical analysis

#### 2.4.1 Univariable-MR analysis

The present study used three methods of MR analysis. The primary analysis estimated causal effect using IVW due to its high statistical power. The IVW method pools the Wald ratio estimates of each SNP on the outcome, providing an overall estimate of causality (24). In consideration of the multiple tests in MR analysis, the Bonferroni correction was applied to correct for type I errors. Therefore, we adjusted the threshold *p*-value for the IVW method to 3.5 × 10^−3^ (0.05/14). A random-effect model was adopted in cases of heterogeneity, while a fixed-effect model was applied when no heterogeneity was present. Moreover, we estimated the causal relationship between anthropometric indicators and risk of sepsis using odds ratios (ORs) with 95% confidence intervals (95% CIs).

To ensure the stability and reliability of MR results, we subsequently performed MR–Egger and weighted-median analyses. MR–Egger was conducted to identify and correct potential pleiotropic effects in order to provide reliable estimates (25). The weighted-median method was used to obtain a solid estimation of the causal effect. This method efficiently controls for type I errors and improves detection ability, even when >50% of the information is derived from invalid IVs (26). The causal-effect estimates of weighted median and MR–Egger (*p* < 0.05) should be consistent with IVW in direction and magnitude. In addition, we used a scatter plot for MR analysis to visualize causal relationships between anthropometric indicators and sepsis risk. To determine the accuracy of causal-effect direction, we performed the MR–Steiger directionality test, with a significance level of *p* < 0.05 suggesting absence of the reverse-causal effect (27).

Due to partial sample overlap, an online calculator was used to examine the risk of bias resulting from potentially weak instruments.^2^ Another online tool was used to assess the statistical power.^3^ All statistical analyses were performed using R software version 4.2.1 (R Foundation for Statistical Computing, Vienna, Austria) with the TwoSampleMR and MR Pleiotropy RESidual Sum and Outlier (MR-PRESSO) packages. Except in IVW analysis, *p* < 0.05 was considered statistically significant.

#### 2.4.2 Sensitivity analysis

We assessed heterogeneity among SNPs using Cochrane’s *Q* test based on IVW and MR–Egger analyses. Significance was set at *p* < 0.05 to determine the presence of potential heterogeneity. However, it is essential to note that the existence of heterogeneity does not necessarily indicate unstable IVW results. We used MR–Egger and MR-PRESSO analyses to identify possible horizontal pleiotropy, the effect of which was represented by *p* < 0.05. MR-PRESSO analysis was used to detect and correct horizontal pleiotropy by deleting outliers and comparing estimates before and after outlier removal (28). Finally, we conducted a leave-one-out analysis by omitting each SNP and testing the remaining ones to identify any outlier SNPs that might confound the causal effect.

#### 2.4.3 MVMR analysis

Due to the relevance of BMI and other obesity-related traits (29), we adopted MVMR analysis to adjust for the genetic association between BMI and each obesity-related indicator. The IVW method served as the primary approach for this analysis.

## 3 Results

### 3.1 Characteristics of genetic IVs

After initial screening, we extracted SNPs associated with obesity-related indicators. None of the identified SNPs were found to be related to sepsis. Supplementary Table S1 provides detailed information on these SNPs. *F* for each SNP ranged from 16 to 5,857, indicating a lower likelihood of weak IVs. Moreover, the range of estimated bias resulting from sample overlap was 0.4–0.7%, suggesting that the MR results were less likely to be affected by bias. The statistical power for all obesity-related indicators and sepsis exceeded 80%, indicating that our study possesses sufficient statistical power to detect potential causal effects.

**Figure 2:**
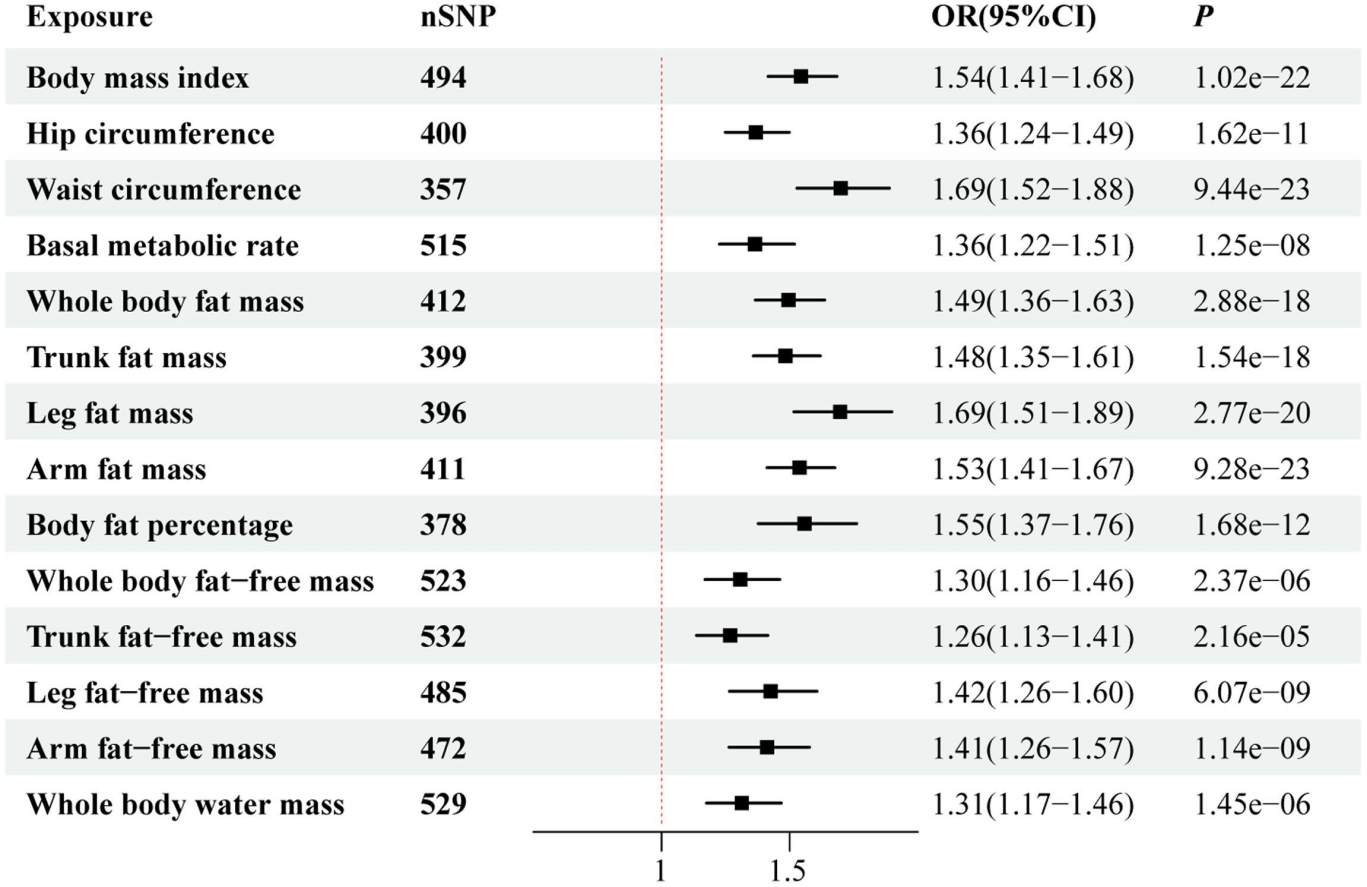
Forest plot visualizing the causal effect of obesity-related anthropometric indicators on sepsis using the inverse variance-weighted (IVW) method.

**Figure 3:**
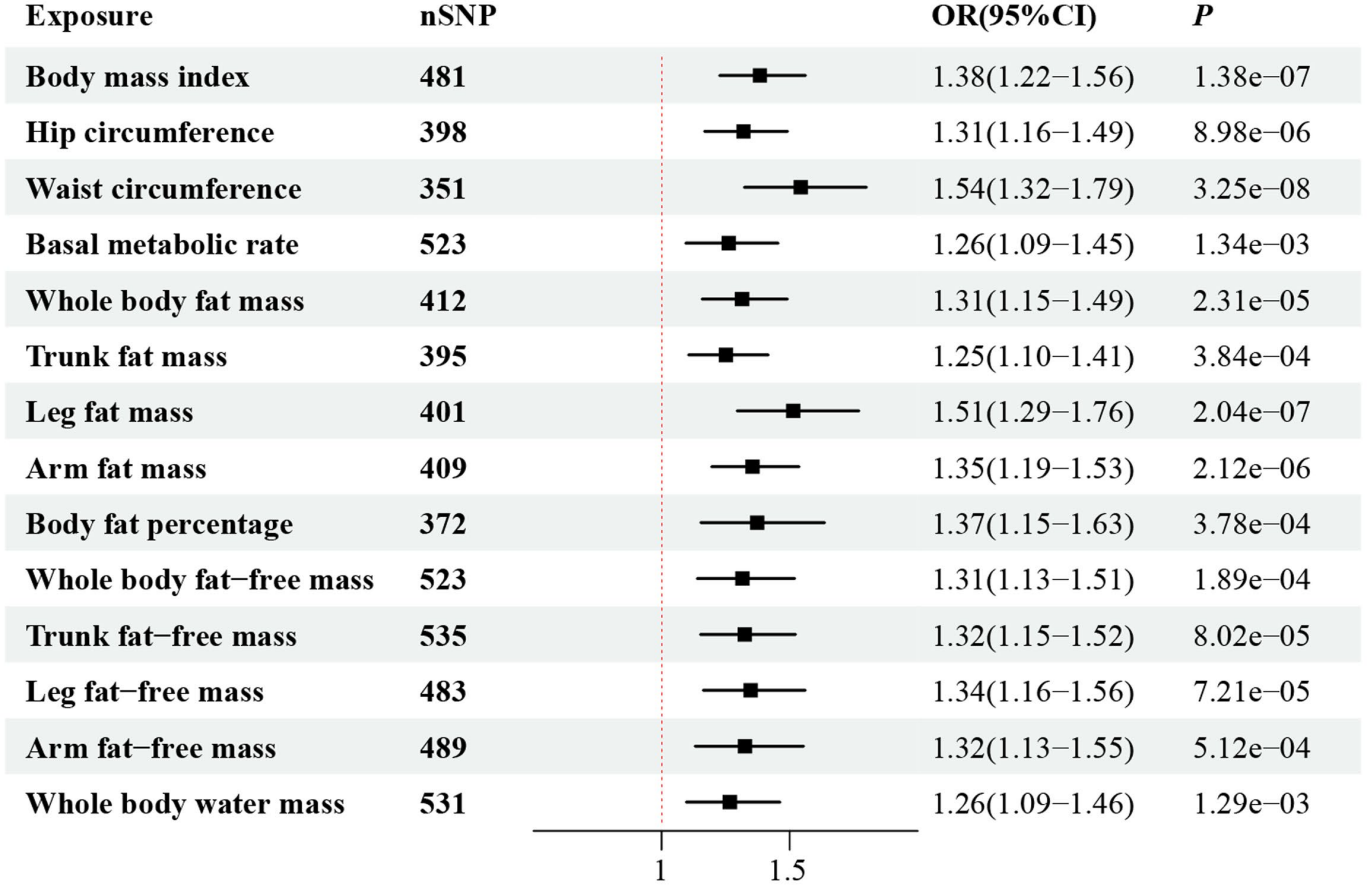
Forest plot visualizing the causal effect of obesity-related anthropometric indicators on sepsis (from the FinnGen cohort) using the IVW method.

**Figure 4:**
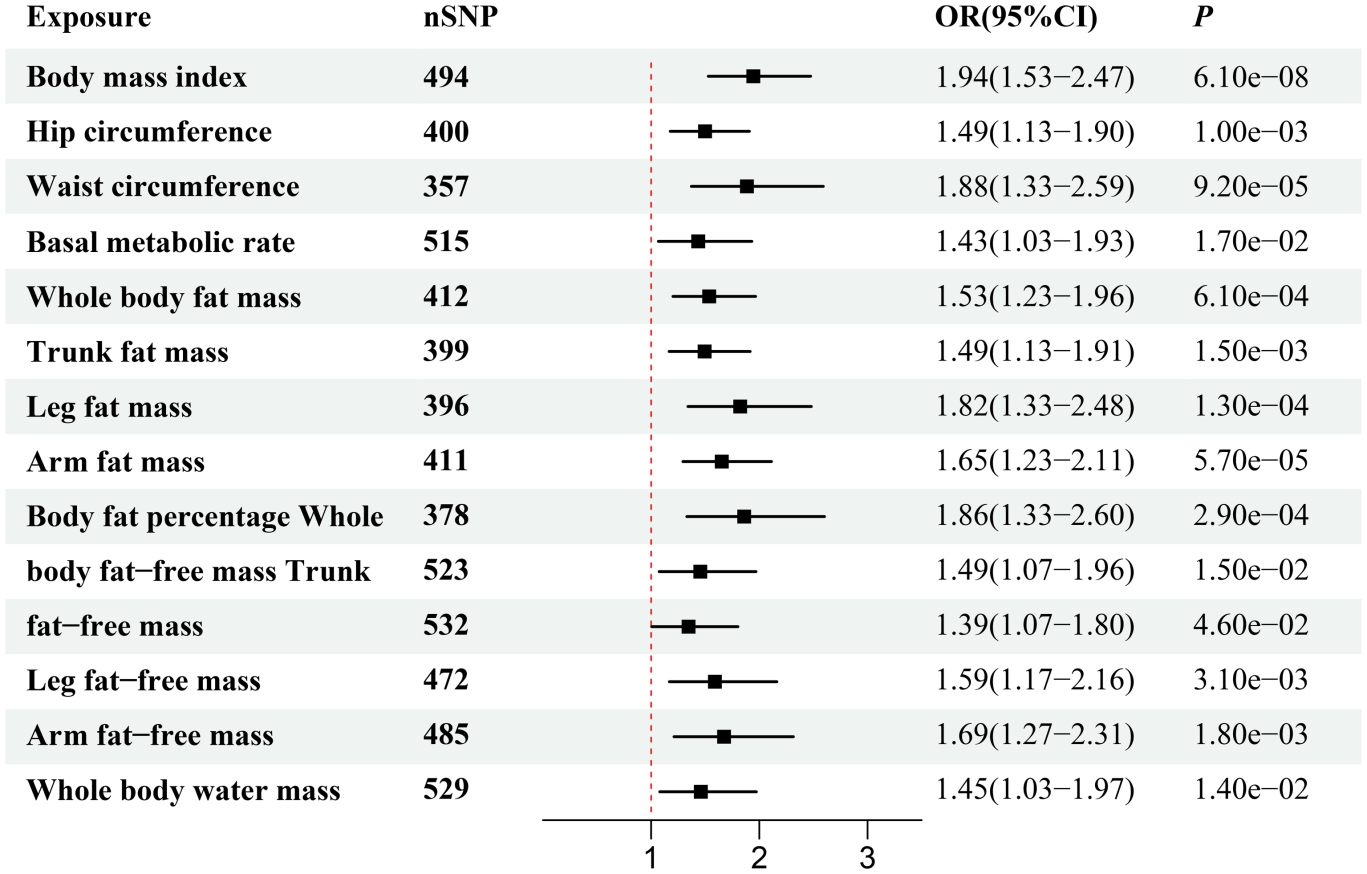
Forest plot visualizing the causal effect of obesity-related anthropometric indicators on critical care sepsis using the IVW method.

**Figure 5:**
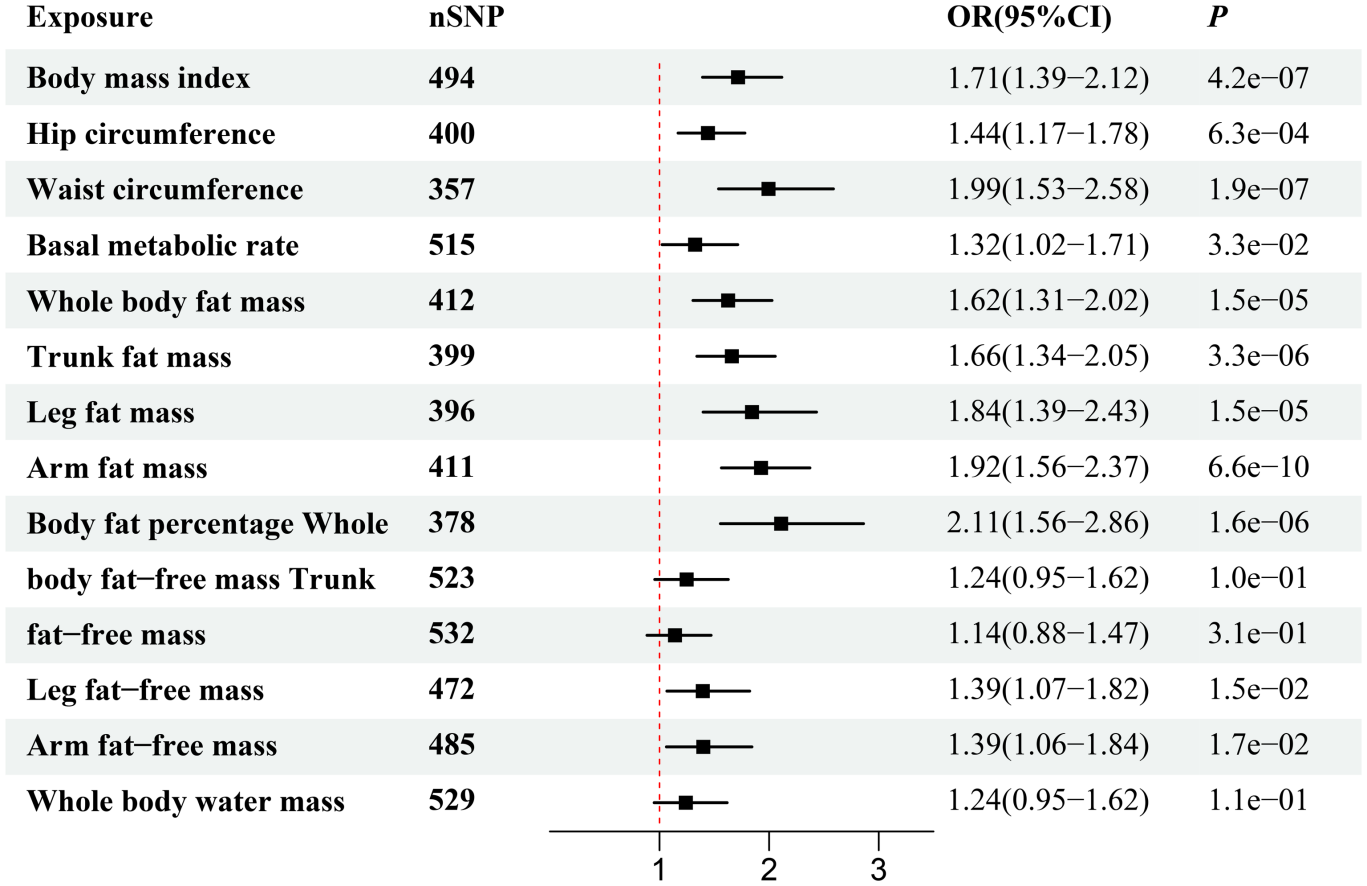
Forest plot visualizing the causal effect of obesity-related anthropometric indicators on 28-day mortality due to sepsis using the IVW method.

Figure 2 illustrates the genetically predicted associations between obesity-related anthropometric indicators and sepsis risk. Specifically, high BMI (OR = 1.54; 95% CI, 1.41–1.68; *p* = 1.02 × 10^−22^), HC (OR = 1.36; 95% CI, 1.24–1.49; *p* = 1.62 × 10^−11^), WC (OR = 1.69; 95% CI, 1.52–1.88; *p* = 9.44 × 10^−23^), BMR (OR = 1.36; 95% CI, 1.22–1.51; *p* = 1.25 × 10^−8^), WBFM (OR = 1.49; 95% CI, 1.36–1.63; *p* = 2.88 × 10^−18^), TFM (OR = 1.48; 95% CI, 1.35–1.61; *p* = 1.54 × 10^−18^), LFM (OR = 1.69; 95% CI, 1.51–1.89; *p* = 2.77 × 10^−20^), AFM (OR = 1.53; 95% CI, 1.41–1.67; *p* = 9.28 × 10^−23^), BFP (OR = 1.55; 95% CI, 1.37–1.76; *p* = 1.68 × 10^−12^), WBFFM (OR = 1.30; 95% CI, 1.16–1.46; *p* = 2.37 × 10^−6^), TFFM (OR = 1.26; 95% CI, 1.13–1.41; *p* = 2.16 × 10^−5^), LFFM (OR = 1.42; 95% CI, 1.26–1.60; *p* = 6.07 × 10^−9^), AFFM (OR = 1.41; 95% CI, 1.26–1.57; *p* = 1.14 × 10^−9^), and WBWM (OR = 1.31; 95% CI, 1.17–1.46; *p* = 1.45 × 10^−6^) were all significantly correlated with an increased risk of sepsis in IVW analysis. We observed similar MR results in the external validation dataset (Figure 3). To further investigate the associations between obesity-related anthropometric indicators and sepsis of varying severities, we expanded our analysis to encompass critical care sepsis and 28-day mortality as outcomes. We observed a positive correlation between elevated levels of all anthropometric indicators and the risk of critical care sepsis. Besides, significant correlations were found for all indicators except WBFFM, TFFM, and WBWM with regards to 28-day mortality caused by sepsis (Figures 4, 5). Detailed MR results from all three methods can be found in Supplementary Table S2. The scatter plots visualize the causal relationship between each anthropometric indicator and sepsis risk (Supplementary Figures S1, S2). We conducted the MR–Steiger test to further explore causal directionality; the results suggested the absence of a reverse-causal effect between anthropometric indicators and sepsis risk (Supplementary Table S3).

### 3.2 Sensitivity analysis

The result of Cochrane’s *Q* test indicated potential heterogeneity in the causal effect between certain anthropometric indicators and sepsis risk (Supplementary Table S4). Specifically, HC (*p* = 0.007), BMR (*p* = 0.026), WBFFM (*p* = 0.004), TFFM (*p* = 0.004), AFFM (*p* = 0.031), and WBWM (*p* = 0.006) exhibited significant heterogeneity. Additionally, the MR–Egger intercept test indicated the absence of horizontal pleiotropy in any MR analysis results (*p* > 0.05; Supplementary Table S5). Furthermore, after removal of outliers, MR-PRESSO correction estimates yielded results consistent with those of primary MR analysis (Supplementary Table S6). Finally, leave-one-out analysis convincingly demonstrated the robustness of the causal relationship between the anthropometric indicators and sepsis risk (Supplementary Table S7).

### 3.3 Causal effects of obesity-related anthropometric indicators on sepsis after adjusting for BMI

**Figure 6:**
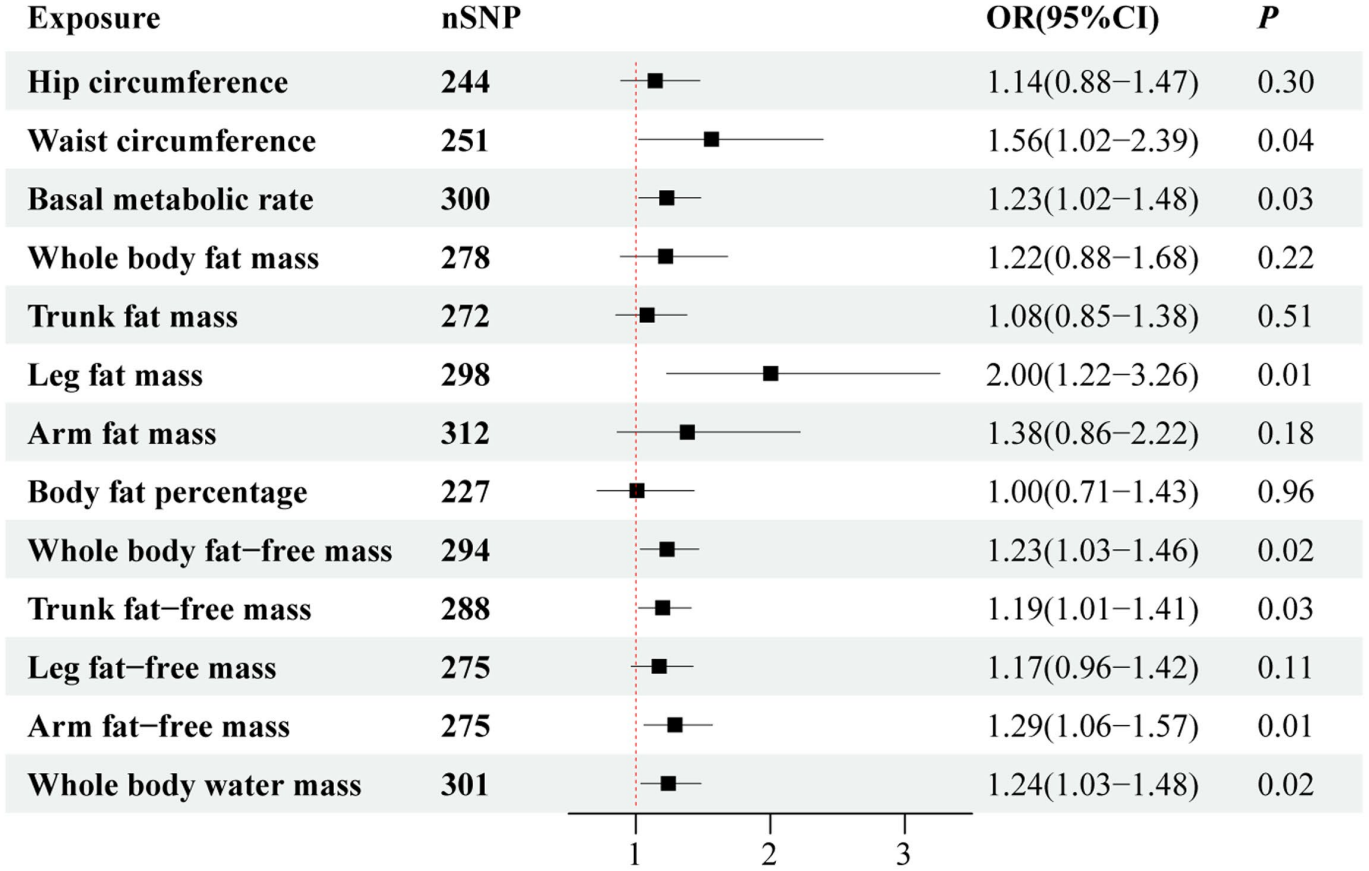
Forest plot visualizing the association between obesity-related anthropometric indicators and sepsis risk after adjusting for body mass index.

Considering the potential relevance of BMI and other anthropometric indicators, we performed MVMR analysis to adjust for the effects of genetically predicted BMI. Thereafter, WC (OR = 1.56; 95% CI, 1.02–2.39; *p* = 0.04), BMR (OR = 1.23; 95% CI, 1.02–1.48; *p* = 0.03), LFM (OR = 2.0; 95% CI, 1.22–3.26; *p* = 0.01), WBFFM (OR = 1.23; 95% CI, 1.03–1.46; *p* = 0.02), TFFM (OR = 1.19; 95% CI, 1.01–1.41; *p* = 0.03), AFFM (OR = 1.29; 95% CI, 1.06–1.57; *p* = 0.01), and WBWM (OR = 1.24; 95% CI, 1.03–1.48; *p* = 0.02) were still significantly correlated with risk of sepsis (Figure 6).

## 4 Discussion

In this MR study, we analyzed associations between obesity-related anthropometric indicators and risk of sepsis using genetic data. We substantiated that the increased values of genetically predicted obesity-related anthropometric indicators, including BMI, HC, WC, BMR, BFP, WBFM, TFM, AFM, LFM, WBFFM, TFFM, AFFM, LFFM, and WBWM, could increase sepsis risk. We then determined an analogous causal relationship in the external validation dataset for sepsis. In relation to critical care sepsis and 28-day mortality attributed to sepsis, the MR analysis yielded consistent findings with the initial analysis. After adjustment for BMI, MVMR analysis revealed that increased WC, BMR, LFM, WBFFM, TFFM, AFFM, and WBWM remained strongly related to a higher risk of sepsis.

Obesity is a significant global health issue, affecting 36.9% of men and 38.0% of women worldwide and contributing to 3.4 million deaths, 3.9% of years of life lost, and 3.8% of disability-adjusted life years (30). Several large cohort studies have demonstrated that obesity, as characterized by elevated BMI, is associated with an increased risk of sepsis or bloodstream infection (5–7). Furthermore, MR studies have identified a causal relationship between genetically predicted BMI and the occurrence of sepsis (8–10). However, BMI alone might not fully capture the complexity of obesity traits because individuals with similar BMIs can exhibit distinct metabolic profiles and differences in body shape and composition (31). Notably, a secondary analysis of the RELIEF study, which enrolled 2,755 participants, demonstrated that WC [OR = 1.45; 95% CI, 1.29–1.63; area under the curve (AUC) = 0.641] outperformed BMI (OR = 1.33; 95% CI, 1.18–1.51; AUC = 0.629) in assessing pre-operative risk of septic complications after elective major abdominal surgery (12). Similarly, a population-based cohort study involving 975 sepsis patients indicated that WC [hazard ratio (HR) = 1.47; 95% CI, 1.20–1.79], but not BMI, was still independently associated with sepsis risk (11). In the present MR study, we found that WC (OR = 1.69; 95% CI, 1.52–1.88) was more significantly associated with sepsis than BMI (OR = 1.54; 95% CI, 1.41–1.68) at the genetic level.

Furthermore, considering the high relevance of BMI and other obesity-related indicators, we applied MVMR analyses to avoid the influence of BMI on the relationship between each anthropometric indicator and sepsis risk. After adjusting for BMI, we found that WC, BMR, LFM, WBFFM, TFFM, AFFM, and WBWM remained strongly related to sepsis risk. These findings highlight the independent associations between these specific obesity-related anthropometric indicators and sepsis risk, suggesting the necessity of more comprehensive anthropometric measures to describe obesity traits in future research.

A reduction in BMR, typically assessed by resting respiratory metabolism and fat-free mass (FFM), is associated with an increased risk of obesity (32). Previous MR studies showed that high BMR might increase the risk of cancer (33) and several cardiovascular diseases (34). However, no evidence showed a causal correlation between BMR and sepsis. Our MR analysis filled this gap, finding a possible causal relationship between high BMR and an increased risk of sepsis. FFM is the lean, fat-devoid component of the body, which serves as a valuable measure of muscle mass (35). A prospective cohort study revealed that neither fat mass nor FFM was correlated with sepsis (36), which contradicted our findings on the causal effects of fat mass and FFM on sepsis risk at the genetic level. It is worth noting that the previous observational study involved 68 post-operative patients in Australia, while our MR study was based on a much larger sample size of nearly 500,000 European volunteers aged between 40 and 69 years. Therefore, we speculate that the discrepancy in results might be attributable to the differences in sample size, inclusion criteria, and potential inherent biases in that observational study, highlighting the need for further research.

Body water mass can be easily obtained through bio-impedance analysis. Obese individuals typically have higher water mass than do those of average weight, and increased water mass is often associated with obesity (37). An observational study found a relevant association between elevated water mass and sleep apnea (38). Furthermore, previous MR studies have indicated that high water mass might increase the risks of atrial fibrillation (39) and sleep apnea (40). However, literature is lacking on the association between water mass and sepsis. Therefore, we conducted the current MR study and found that high water mass might contribute to increased sepsis risk. This causal relationship should be further investigated in future large-scale observational studies and RCTs.

The underlying mechanism linking obesity-related anthropometric indicators and sepsis risk has been explored in several studies. Excessive fat accumulation in obesity results in the release of inflammatory mediators from adipose tissues, promoting a pro-inflammatory state and oxidative stress (OS) (41, 42). Skeletal muscle, identified as an endocrine organ, produces and releases various cytokines, particularly interleukin-6 (43). Higher FFM is associated with increased inflammation, OS, and endothelial dysfunction compared with lower FFM (44). Moreover, elevated BMR reflects an increase in energy production, leading to mass generation of reactive oxygen species (ROS) (45). Abnormal accumulation of ROS triggers OS and the production of pro-inflammatory cytokines, which play pivotal roles in the development of sepsis (46). The prolonged presence of these pro-inflammatory factors can induce systemic inflammation and immune cell alterations in distant organs, leading to multi-organ dysfunction (47). Further investigation is warranted to understand the potential biological mechanism underlying the association between obesity-related anthropometric indicators and sepsis risk.

The present study had several strengths. Its primary advantage was the use of MR, which helped minimize confounders and provided a causal inference (48). In addition, unlike previous studies that primarily relied on BMI, our MR study incorporated comprehensive obesity-related anthropometric indicators to clarify the effects of body size and composition on sepsis. Furthermore, similar results were obtained from an external validation dataset in the FinGenn cohort, enhancing the generalizability and robustness of our findings. Finally, the predominance of participants with European ancestry reduced bias related to population architecture.

Despite the above advantages, there are limitations to this study that should be acknowledged. First, there was a partial sample overlap between obesity-related anthropometric indicators and sepsis risk. However, in large datasets such as the UK Biobank, sample overlap exerts only a minimal influence on results (49). We also quantified relative bias resulting from sample overlap (0.4–0.7%), which was unlikely to have significantly affected our overall conclusion. Second, body composition differed by age and gender, but we could not further perform stratified analysis because the GWAS data used in this study consisted of summary-level statistics. Furthermore, it is worth noting that the sepsis diagnoses in our study were based on ICD-10 codes A02, A39, A40, and A41 rather than the Sepsis-3 definitions (50), potentially impacting the precision of our sepsis subtype classification. To ensure enhanced accuracy of study findings and alignment with current clinical practices, future research should consider adopting diagnostic codes that align with the Sepsis-3 criteria. Third, due to our restriction of participants to those of European descent, caution should be exercised when interpreting the results. Again, further research is required to comprehensively investigate the causal effect of obesity-related anthropometric indicators on sepsis, taking into account individual demographic characteristics across populations of diverse descent.

## 5 Conclusion

This MR study provided robust evidence supporting a causal role of obesity-related anthropometric indicators in risk of sepsis. Specifically, elevated levels of these indicators were associated with an increased risk of sepsis. These findings enhance our understanding of how obesity affects sepsis risk. Further research is needed to validate these findings and investigate the underlying mechanisms.

## Data Availability

The original contributions presented in the study are included in the article/Supplementary material, further inquiries can be directed to the corresponding author.

## Footnotes

^1^ https://gwas.mrcieu.ac.uk/

^2^ https://sb452.shinyapps.io/overlap

^3^ https://cnsgenomics.shinyapps.io/mRnd/
