# Supplementary material for: Causal relationships between obesity-related anthropometric indicators and sepsis risk: a Mendelian-randomization study
Source: Front Nutr. 2024 Sep 23;11:1433754. doi: 10.3389/fnut.2024.1433754 (PMC11449881; doi:10.3389/fnut.2024.1433754)

Supplementary Figure 1. Scatter plot of the causal relationships between body anthropometric indicators and sepsis using different MR methods. (A) Causal estimates for body mass index on sepsis; (B) Causal estimates for hip circumference on sepsis; (C) Causal estimates for whole body fat mass on sepsis; (D) Causal estimates for body fat percentage on sepsis; (E) Causal estimates for leg fat-free mass on sepsis; (F) Causal estimates for basal metabolic rate on sepsis; (G) Causal estimates for arm fat-free mass on sepsis; (H) Causal estimates for waist circumference on sepsis; (I) Causal estimates for whole body fat-free mass on sepsis; (J) Causal estimates for trunk fat-free mass on sepsis; (K) Causal estimates for trunk fat mass on sepsis; (L) Causal estimates for arm fat mass on sepsis.; (M) Causal estimates for whole body water mass on sepsis; (N) Causal estimates for leg fat mass on sepsis. The slopes of the line represent the causal association for different methods. The light blue line represents the Inverse variance weighted estimate, the dark blue line represents the MR Egger, the light green line represents the weighted median estimate.

Supplementary Figure 2. Scatter plot of the causal relationships between body anthropometric indicators and septicemia using different MR methods. (A) Causal estimates for body mass index on septicemia; (B) Causal estimates for hip circumference on septicemia; (C) Causal estimates for whole body fat mass on septicemia; (D) Causal estimates for body fat percentage on septicemia; (E) Causal estimates for leg fat-free mass on septicemia; (F) Causal estimates for basal metabolic rate on septicemia; (G) Causal estimates for arm fat-free mass on septicemia; (H) Causal estimates for waist circumference on septicemia; (I) Causal estimates for whole body fat-free mass on septicemia; (J) Causal estimates for trunk fat-free mass on septicemia; (K) Causal estimates for trunk fat mass on septicemia; (L) Causal estimates for arm fat mass on septicemia; (M) Causal estimates for whole body water mass on septicemia; (N) Causal estimates for leg fat mass on septicemia. The slopes of the line represent the causal association for different methods. The light blue line represents the Inverse variance weighted estimate, the dark blue line represents the MR Egger, the light green line represents the weighted median estimate.

Supplementary Figure 1

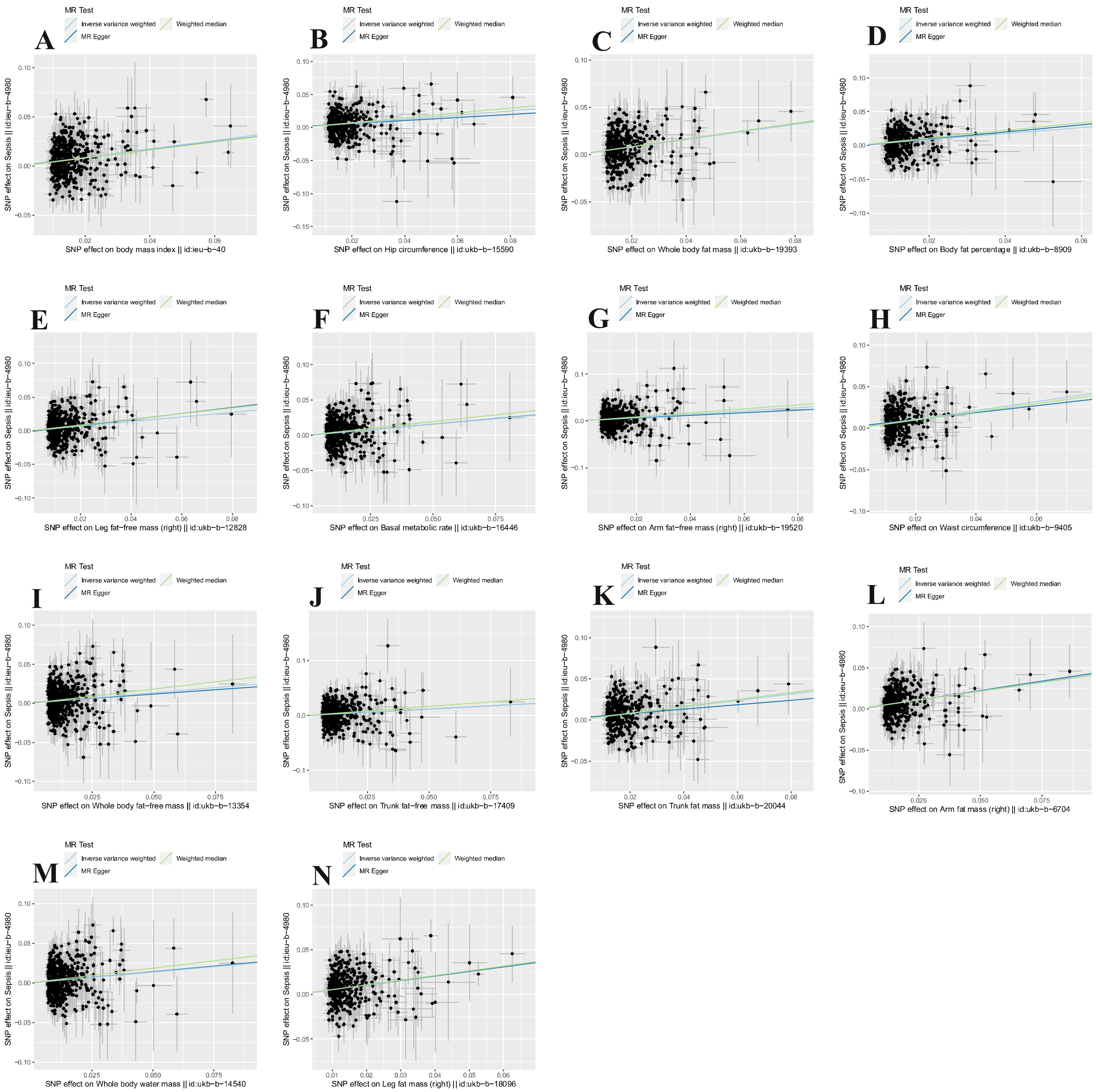

Supplementary Figure 2

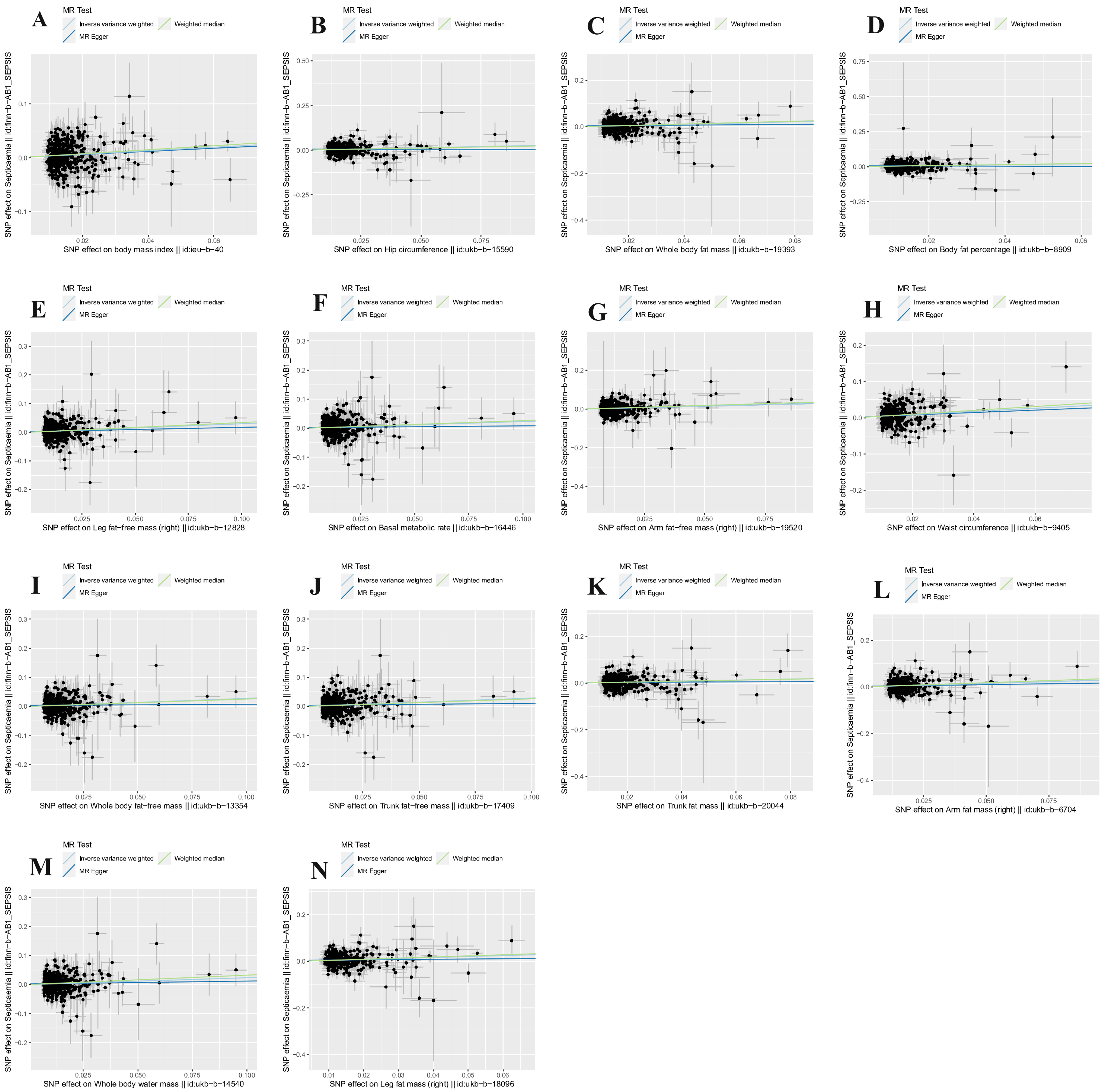

Supplement: Supplementary file 1 [file Image_1.PDF]
